# Integrated Transcriptome Analysis and Single-Base Resolution Methylomes of Watermelon (*Citrullus lanatus*) Reveal Epigenome Modifications in Response to Osmotic Stress

**DOI:** 10.3389/fpls.2021.769712

**Published:** 2021-11-29

**Authors:** Fangming Zhu, Mingyan Li, Manwen Yan, Fei Qiao, Xuefei Jiang

**Affiliations:** ^1^Key Laboratory for Quality Regulation of Tropical Horticultural Plants of Hainan Province, College of Horticulture, Hainan University (HNU), Haikou, China; ^2^Key Laboratory of Tropical Agritourism in Greenhouse of Haikou, College of Horticulture, Hainan University (HNU), Haikou, China; ^3^Key Laboratory of Crop Gene Resources and Germplasm Enhancement in Southern China, Ministry of Agriculture, Chinese Academy of Tropical Agricultural Sciences, Danzhou, China; ^4^Tropical Crops Genetic Resources Institute, Chinese Academy of Tropical Agricultural Sciences, Danzhou, China

**Keywords:** *Citrullus lanatus*, osmotic stress, transcriptome analysis, whole genome bisulfite sequencing, DNA methylation

## Abstract

DNA methylation plays an important role against adverse environment by reshaping transcriptional profile in plants. To better understand the molecular mechanisms of watermelon response to osmotic stress, the suspension cultured watermelon cells were treated with 100mM mannitol, and then a methylated cytosines map was generated by whole genome bisulfite sequencing (WGBS). Combined with transcriptome sequencing, the effects of osmotic stress on differentially methylated expressed genes (DMEGs) were assessed. It was found that genes related to plant hormone synthesis, signal transduction, osmoregulatory substance-related and reactive oxygen species scavenging-related enzyme could rapidly respond to osmotic stress. The overall methylation level of watermelon decreased after osmotic stress treatment, and demethylation occurred in CG, CHG, and CHH contexts. Moreover, differentially methylated expressed genes (DMEGs) were significantly enriched in RNA transport, starch and sucrose metabolism, plant hormone signal transduction and biosynthesis of secondary metabolites, especially in biosynthesis of osmolytes synthase genes. Interestingly, demethylation of a key enzyme gene *Cla014489* in biosynthesis of inositol upregulated its expression and promoted accumulation of inositol, which could alleviate the inhibition of cell growth caused by osmotic stress. Meanwhile, a recombinant plasmid pET28a-*Cla014489* was constructed and transferred into *Escherichia coli* BL21 for prokaryotic expression and the expression of *Cl*MIPS protein could improve the tolerance of *E. coli* to osmotic stress. The effect of methylation level on the expression properties of inositol and its related genes was further confirmed by application of DNA methylation inhibitor 5-azacytidine. These results provide a preliminary insight into the altered methylation levels of watermelon cells in response to osmotic stress and suggest a new mechanism that how watermelon cells adapt to osmotic stress.

## Introduction

With the continuous exploration of molecular mechanisms, it has been discovered that some biological phenomena do not exactly conform to genetic inheritance laws. The emergence of epigenetics has served as a good complement to elaborate some biological processes (Waddington, 1942; Bird, 2007). Among them, DNA methylation is the earliest discovered and most intensively studied epigenetic modification (Duan et al., 2018). Increasing literature has demonstrated that DNA methylation plays an important role in regulating gene expression, silencing transposons, and maintaining genome integrity (Tompa et al., 2002; Hoa et al., 2016). In plants, DNA methylation patterns and levels are diverse across species and can typically occur at symmetric CG and CHG sites and asymmetric CHH sites where H represents A, C, or T (Cao and Jacobsen, 2002; Henderson and Jacobsen, 2007). They can all be established by Domains Rearranged Methyltrans-ferase 2 (DRM2) *via* the RNA-directed DNA methylation pathway and maintained by different methylation transferases (Law and Jacobsen, 2010; Stroud et al., 2014). DNA methylation is a reversible process, which can be hydrolyzed to cytosine and water by demethylase, or 5mC can be removed from the phosphodiester bond skeleton by DNA glycosylase (Yuan et al., 2020). It was found that most DNA methylation modified genes in plants undergo transcriptional changes in response to adversity stress, suggesting that complex DNA methylation patterns are directly related to stress response and ultimately affect gene expression (Lu et al., 2017; Xu et al., 2018a).

Being sessile in nature, plants have evolved complex mechanisms to sense external signals during their long evolutionary and adaptive processes in order to respond optimally to environmental changes (Wang et al., 2016). Among them, epigenetic mechanism is one of the important mechanisms discovered in recent years to regulate plant responses to external stresses. As the role of DNA methylation in plant growth and stress resistance becomes clearer, efficient and accurate DNA methylation detection methods are essential for gaining insight into the mechanisms of DNA methylation in plant growth, development, and stress adaption. Various complex molecular techniques have been used previously, including methylation sensitive amplified polymorphism (MSAP), enzyme linked immunosorbent assay (ELISA), and methylated DNA immunoprecipitation-sequencing (MeDIP-seq); they have been used to study abiotic stress-induced DNA methylation changes in different plant species (Correia et al., 2013; Stelpflug et al., 2014). These studies provided evidences regarding the importance of such epigenetic processes in controlling plant responses to adversity. With the development of high-throughput sequencing technology, whole genome bisulfite sequencing (WGBS) technology based on the bisulfite transformation method is currently the most advanced and direct method for analyzing dynamic changes in DNA methylation (Liu et al., 2017). This technique has the advantages that it can resolve genome-wide DNA methylation patterns of species with high throughput, high resolution, and single nucleotide resolution. So far, single-base resolution of DNA methylation groups of several species has been accomplished by applying this technique, including *Arabidopsis*, rice, maize, soybean, tomato, and poplar (Chodavarapu et al., 2012; Gent et al., 2013; Song et al., 2013; Zhong et al., 2013; Liang et al., 2014; Zhang et al., 2015). The detection of differential methylation patterns in response to water stress based on the construction of single-base resolution methylation groups in watermelon has not been reported.

Watermelon (*Citrullus lanatus*), as an important fruit type economic horticultural crop, often faces a series of abiotic adversities in production, such as drought, salinity and cold damage, which could produce osmotic stress and affect the quality, yield and even the development of the entire watermelon industry. Therefore, understanding the regulatory mechanisms in adversities and identifying resistance genes in tolerant plants are of great significance for guiding plant genetic resource management and plant breeding. With the continuous development of gene editing technologies, great progress has been made in agronomic trait improvement, but the genetic information of resistant plants is still poorly understood (Hua et al., 2019). Watermelon, a drought-tolerant species originating from the Sahara Desert, is an ideal model plant for studying water stress. The completion of whole genome sequencing of watermelon and the rapid development of high-throughput sequencing technologies provide unprecedented opportunities to comprehensively examine epigenetic modifications and correlate them with gene expression (Guo et al., 2013). Compared to plant materials, suspension cells have been used as an important model for studying stress-related experiments due to their good dispersion, high homogeneity and rapid growth (Li et al., 2018). Here, we generated a genome-wide DNA methylation map of watermelon cells after osmotic stress treatment with high coverage. The study was designed to collect data and provide insight into three questions: (i) to determine genome-wide watermelon methylome levels; (ii) to analyze changes in watermelon methylation associated with osmotic stress, and (iii) to evaluate the relationship between the changes of methylome and osmotic stress-related gene expression. This study provides a possibility for further understanding the effects of differentially methylated regions on gene expression and osmotic stress, and refers a reference for exploring the basis of resistance in watermelon.

## Materials and Methods

### Plant Material and Osmotic Stress Treatment

Suspension cells of *Citrullus lanatus* preserved at the Tropical Crop Germplasm Research Institute, Chinese Academy of Tropical Agricultural Sciences (CATAS), were used in this study, and their establishment and maintenance details were as described in our previous publication (Xu et al., 2018b).

After 3–4days of sub-cultivation, cells were treated with D-mannitol (100mM) for 2h and 4h, and then the treated cells were centrifuged at 4,000rpm for 5min. The supernatant was discarded and cells frozen in liquid nitrogen, stored at −80°C for DNA and RNA extraction. The untreated (0h) was used as the control, with three biological replicates set for each treatment.

### RNA Extraction and Transcriptome Sequencing

Total RNA was extracted with the RNAprep Pure Plant Kit (TIANGEN) according to the manufacturer’s instructions. The purity, concentration and integrity of the RNA were analyzed by NanoPhotometer® spectrophotometer (IMPLEN, CA, USA), Qubit® 2.0 Fluorometer (Life Technologies, CA, USA), Agilent 2,100 bioanalyzer (Agilent Technologies, CA, USA). After the samples were tested and qualified, cDNA libraries were constructed by using NEBNext® UltraTM RNA Library Prep Kit for Illumina® (NEB, USA) according to the NEB common library construction method (Parkhomchuk et al., 2009), and then RNA-Seq sequencing analysis was completed by Illumina Hi-Seq platform (Novogene, Beijing, China). The raw reads were filtered to remove reads with adapter, N (N means base information cannot be determined) and low-quality reads reduced the interference of analysis caused by invalid data. The clean reads were compared with the Watermelon Genome Database (http://www.icugi.org/cgi-bin/ICuGI/index.cgi) (Guo et al., 2013). *p*-value <0.05 and |log_2_(FoldChange)|>1 were selected as criteria to screen differentially expressed genes by using DESeq2 R package (1.16.1), and GO and KEGG analyses of DEGs were performed by GO-Seq R package and KOBAS software (Mao et al., 2005; Young et al., 2010). GO terms or KEGG pathways with a corrected p-value <0.05 were considered to be significantly enriched.

### DNA Extraction and BS-SEQ Library Construction

Due to the similarity of gene expression patterns between the 2-h and 4-h treatments of osmotic stress, control and mannitol-treated 4h cells were selected for methylation analysis. Three biological replicates were set for each treatment. Total genomic DNA was extracted according to the CATB method. The purity, concentration, and integrity of DNA were checked by the NanoPhotometer® spectrophotometer (IMPLEN, CA, USA), Qubit® DNA Assay Kit in Qubit® 2.0 Fluorometer (Life Technologies, CA, USA) and 1% agarose gel electrophoresis. After that, DNA of three repetitions was mixed in equal amounts for library construction.

BS-SEQ library construction was performed according to previous studies (Lu et al., 2017; Xu et al., 2018a). A total of 5.2μg of genomic DNA doped with 26ng Lambda DNA (negative control) was sonicated to 200–300bp with Covaris S220, followed by end repair and adenylation. Cytosine methylated barcodes were ligated to the sonicated DNA fragments according to the manufacturer’s instructions. These DNA fragments were treated twice with bisulfite using the EZ DNA Methylation-GoldTM Kit (Zymo Research), and the resulting single-strand DNA fragments were PCR amplificated using KAPA HiFi HotStart Uracil + ReadyMix PCR Kit. Library concentration was quantified by Qubit® 2.0 Fluorometer (Life Technologies, CA, USA) and quantitative PCR, and the size of the insert fragments was determined on an Agilent Bioanalyzer 2,100 system. Library preparations were sequenced on the Illumina Hiseq 2,500 platform (Novogene, Beijing, China).

### Bisulfite Sequencing Data Analysis

Raw bisulfite sequencing data were filtered by removing aptamer sequences, low-quality reads, and contamination. The clean data were compared to the Watermelon Genome Database using Bismark software (0.16.3). After removing duplicate reads, the mapped reads were combined and cytosine methylation levels were calculated for each methylation (Lister et al., 2013). Differential methylation regions (DMRs) were identified using DSS software. GO and KEGG analyses were performed for genes associated with DMR using the GO-Seq R package and KOBAS software (Mao et al., 2005; Young et al., 2010), setting a corrected *p*-value <0.05 as the threshold value.

### 5-Azacytidine Treatment

After 3–4days of sub-cultivation, 100mM 5-aza was added to a final concentration of 100μM. And 1 M mannitol was added to a final concentration of 100mM after 12h of incubation at 25°C, 150rpm on a shaker. 1.8ml of cells was aspirated in 2-mL centrifuge tubes at 0h, 2h and 4h of stress treatment, respectively, for the quantification of gene expression, and 0.5g of filtered cells was weighed at 24h of osmotic stress induction for the determination of inositol content.

The quantification of gene expression was determined by quantitative real-time RT-PCR (RT-qPCR) which was performed as shown by Xu et al. (2018b). *18S rRNA* was selected as the reference gene, and the gene expression was quantified by 2^-ΔΔCT^ method (Liu et al., 2014). The primers were designed using Beacon Designer 7 software and are listed in Supplementary Table S1. Each gene was biologically repeated three times, with three multiple wells per repeat.

To measure the content of inositol, 0.5g of fresh weight cells was mixed with 1ml of 50% methanol, 2 steel beads (d=4mm) were added and ground for 90s using a freeze grinder (Shanghai, China) at 75Hz. The supernatant was filtered through a 0.22μm microporous membrane after centrifugation (8,000rpm, 10min) and analyzed using ultraperformance liquid chromatography coupled to mass spectrometry (UPLC–MS). The separation was performed on an Agilent infinity 1,260 (Agilent Technologies) device equipped with an Agilent Zorbax Hilic Plus column (100mm×3.0mm, 1.8μm) maintained at 30°C. The mobile phase was acetonitrile: water (20:80, v/v), and the flow rate was set to 0.3ml·min^−1^, with the injection volume of 2μl.

Inject into the mass analyzer using the electrospray ionization (ESI) probe inlet. Ions were generated and focused using an ESI voltage of 3.7 KV; sheath gas (nitrogen) flow rate, 40 arb; auxiliary gas (nitrogen) flow rate, 15 arb; capillary temperature, 320°C. The signal was achieved in the negative ion mode of ESI.

A concentration of 0.4mg·mL^−1^ was obtained by adding 50% methanol to inositol standard powder (≥98%, Shanghai Yuanye Biotechnology Co., Ltd., China). This inositol standard solution was diluted to five calibration concentrations and measured under the UPLC-MS conditions described above. Inositol was quantified using peak areas based on calibration curves for different concentrations of standards. Y=21386.5X+61,163, R^2^=0.9994, with X inositol concentration, and Y peak area.

### Determination of Cell Growth and Cell Damage

Packed cell volume (∆PCV) determination method was shown as Ismail et al. (2012). MDA (Malondialdehyde) content was determined by Malondialdehyde Kit (Kemin, Suzhou, China) according to the manufacturer’s instructions.

### Prokaryotic Expression of *Cl*MIPS

Total RNA was extracted using the AxyPrep Multisource Total RNA Miniprep Kit (Axygen, Hangzhou, China) and transcribed into cDNA by the PrimeScriptTM RT reagent Kit with gDNA Eraser Kit (Takara, Dalian, China). The primer was designed according to the CDS sequence, and PCR amplification was performed using Primer STAR max enzyme (Takara). The amplification products were ligated to the pET-28a cloning vector and transformed into *Escherichia coli* BL21. Single colonies were randomly selected for PCR identification, and the positive plasmids were sent to Sangon Biotech (Shanghai) Co. for sequencing analysis.

The IPTG-induced bacterial broth was diluted with LB liquid medium in gradients of 1, 10^−1^, 10^−2^, 10^−3^, and 10^−4^. 3μl of each broth was added dropwise to the medium containing 600mM mannitol (LB as the control). The growth and survival of recombinant strain pET-28a-*Cla014489* and control strain pET-28a were observed after cultured at 37°C for 12h (Cheng et al., 2016). For measurement of the time course of growth under osmotic conditions, the induced bacterial solution was inoculated into LB liquid medium with 600mM mannitol, and samples were taken at an interval of 2h and a total of 12h. The optical density was measured using spectrophotometer (Benchmark Plus, BIO-RAD, USA).

### Determination of the Content of Plant Hormones and Osmoregulatory Substances

GA, JA, SA, and ABA were analyzed using UPLC-MS with an Agilent Infinitylab Poroshell 120 EC-C18 column (100mm×2.1mm, 2.7μm) maintained at 30°C. Hormones were separated using H_2_O containing 0.1% formic acid (solvent A) and methanol (solvent B) at a flow rate of 0.3ml·min^−1^ under the following conditions: 98% A solvent for 0–2min; 98–45% A solvent for 2–2.1min; 45–5% A solvent for 2.1–3min; 5–35% A solvent for 3–5min; 35–98% A solvent for 5–8min and 98% A solvent for 8–10min. ESI voltage of 3.7 KV; sheath gas (nitrogen) flow rate, 40 arb; auxiliary (nitrogen) flow rate, 15 arb; capillary temperature, 320°C. Signals were achieved in the positive ion mode of ESI. Plant hormones contents were quantified by peak area based on calibration curves for various concentrations of standards. Ethylene was determined by gas chromatography–mass spectrometry (GC–MS) as described by López-Ráez et al. (2010). The methods for the determination of trehalose and betaine content were shown as Garg et al. (2007) and Xu et al. (2018b) respectively. Three biological replicates were used for all analyses of changes in content.

### Data Analysis

Data were summarized and organized using Excel 2010 (Microsoft Corporation, USA) and plotted using TB tools software (Chen et al., 2020).

## Results

### Analysis of DEGs in Watermelon Cells Under Osmotic Stress

**Figure 1:**
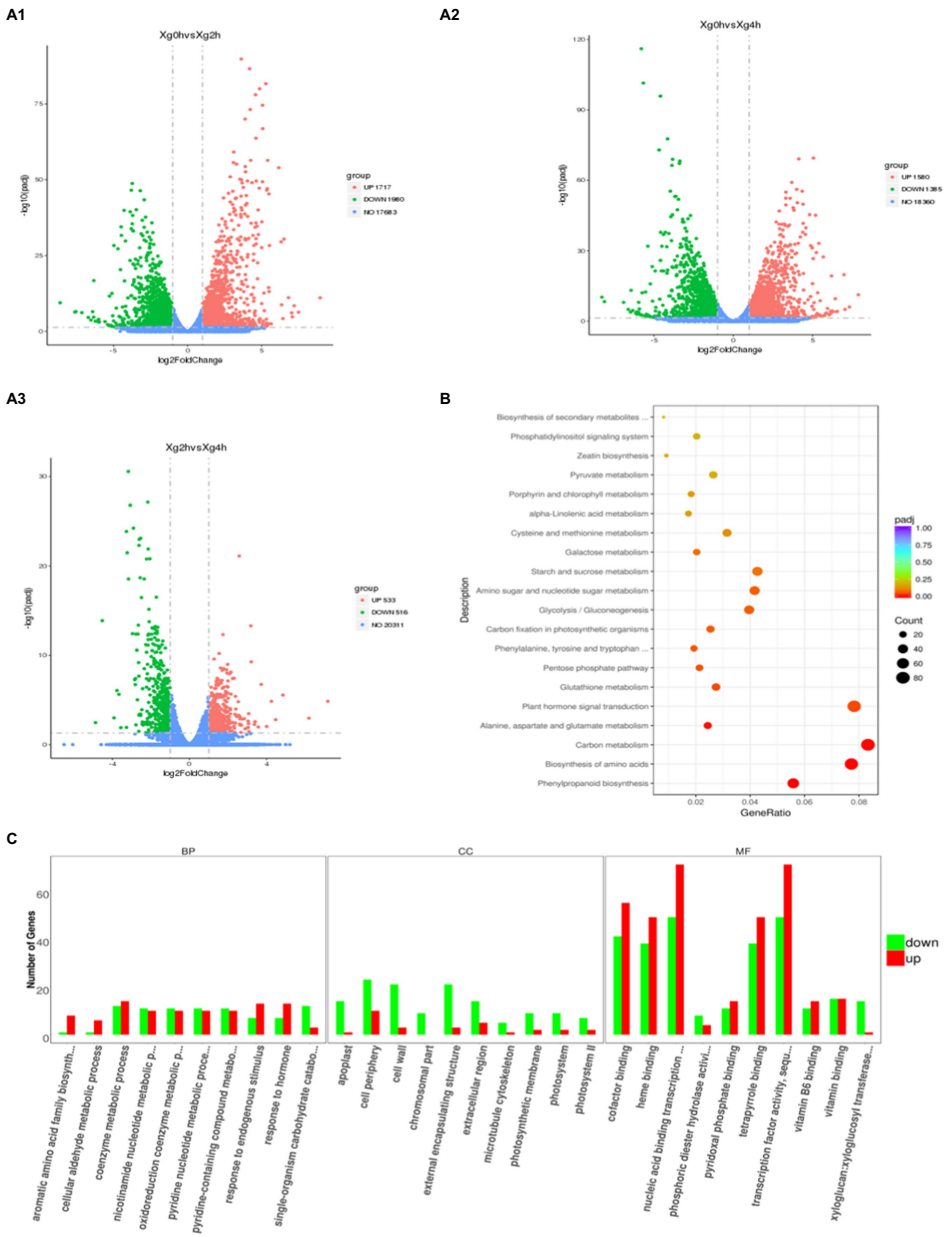
Transcriptomic profiling of watermelon cells in response to osmotic stress. **(A)** Volcano maps of DEGs between different treatments. Red dots indicate up-regulated genes, green dots indicate down-regulated genes, and blue dots indicate out-of-threshold genes. **(B)** KEGG enrichment analysis of DEGs at 0h and 4h. The size of the circles represents the gene number and the color represents the q value. The same below. **(C)** GO enrichment analysis of DEGs at 0h and 4h. The x-axis represents three domains of GO while y-axis represents the gene numbers within each pathway and processes.

Ilumina HiSeq platform generated a total of 43,632,402, 49,495,613 and 48,639,817 clean reads from the control and stress treatments at 2h and 4h, respectively (Table 1). The clean reads were compared with the Watermelon Genome Database (Guo et al., 2013) to screen for differentially expressed genes (DEGs), and a total of 3,697 DEGs were observed in the 2h compared with the control (Figure 1A1), of which 1,717 genes were up-regulated and 1,980 genes were down-regulated. A total of 2,960 DEGs were observed in the 4-h treatment (Figure 1A2), of which 1,580 genes were up-regulated and 1,385 genes were down-regulated. While comparing the 2h against 4-h treatments, 533 DEGs were up-regulated and 516 DEGs were down-regulated (Figure 1A3). A lower number of DEGs indicates that the gene expression patterns of watermelon suspension cells at 2h and 4h were similar after osmotic stress.

**Table 1:** Transcriptome sequencing quality assessment.

Sample	Raw reads	Clean reads	Q20 (%)	Q30 (%)	GC content (%)	Total map (%)
0h_1	44,829,406	43,484,122	98.04	94.36	43.06	41,157,017 (94.65%)
0h_2	47,109,094	44,900,034	98.13	94.48	43.55	43,077,949 (95.94%)
0h_3	54,134,846	52,576,824	98.22	94.70	43.32	50,254,934 (95.58%)
2h_1	47,233,256	43,632,402	98.20	94.67	43.44	41,972,987 (96.2%)
2h_2	55,182,038	53,740,082	97.05	91.91	43.20	51,410,125 (95.66%)
2h_3	52,413,096	51,114,356	97.14	92.04	43.90	49,317,247 (96.48%)
4h_1	49,648,978	48,734,462	96.96	91.76	42.82	45,843,181 (94.07%)
4h_2	50,816,168	49,738,796	98.38	95.01	43.84	48,194,249 (96.89%)
4h_3	48,585,622	47,446,194	98.24	94.60	44.15	45,805,217 (96.54%)

GO enrichment analysis of 2,960 DEGs between control and 4-h treatments was performed using the GO-Seq R package (Figure 1C). In the cellular component (CC), DEGs were involved in biological functions mainly in the cell periphery, cell wall and intracellular membrane-bound organelle fractions. The greatest number of DEGs were enriched in molecular functions (MF), among which transferase activity, transcription factor activity, hydrolase activity, and transmembrane transporter protein activity were the most. In terms of biological processes (BP), they were mainly enriched in the entries of response to stimuli, carbohydrate metabolism, transmembrane transport, biosynthesis of organic nitrogen compounds, and ion transport. These DEGs, after KEGG enrichment analysis, were mainly enriched in carbon metabolism, plant hormone signal transduction, biosynthesis of amino acids, phenylpropanoid biosynthesis, starch and sucrose metabolism, amino sugar and nucleotide sugar metabolism, glycolysis/gluconeogenesis, protein processing in endoplasmic reticulum, plant-pathogen interaction, endocytosis and MAPK signaling pathway metabolic pathways (Figure 1B).

### Screening and Characterization of DEGs Associated With Osmotic Stress Adaptation in Watermelon Cells

**Figure 2:**
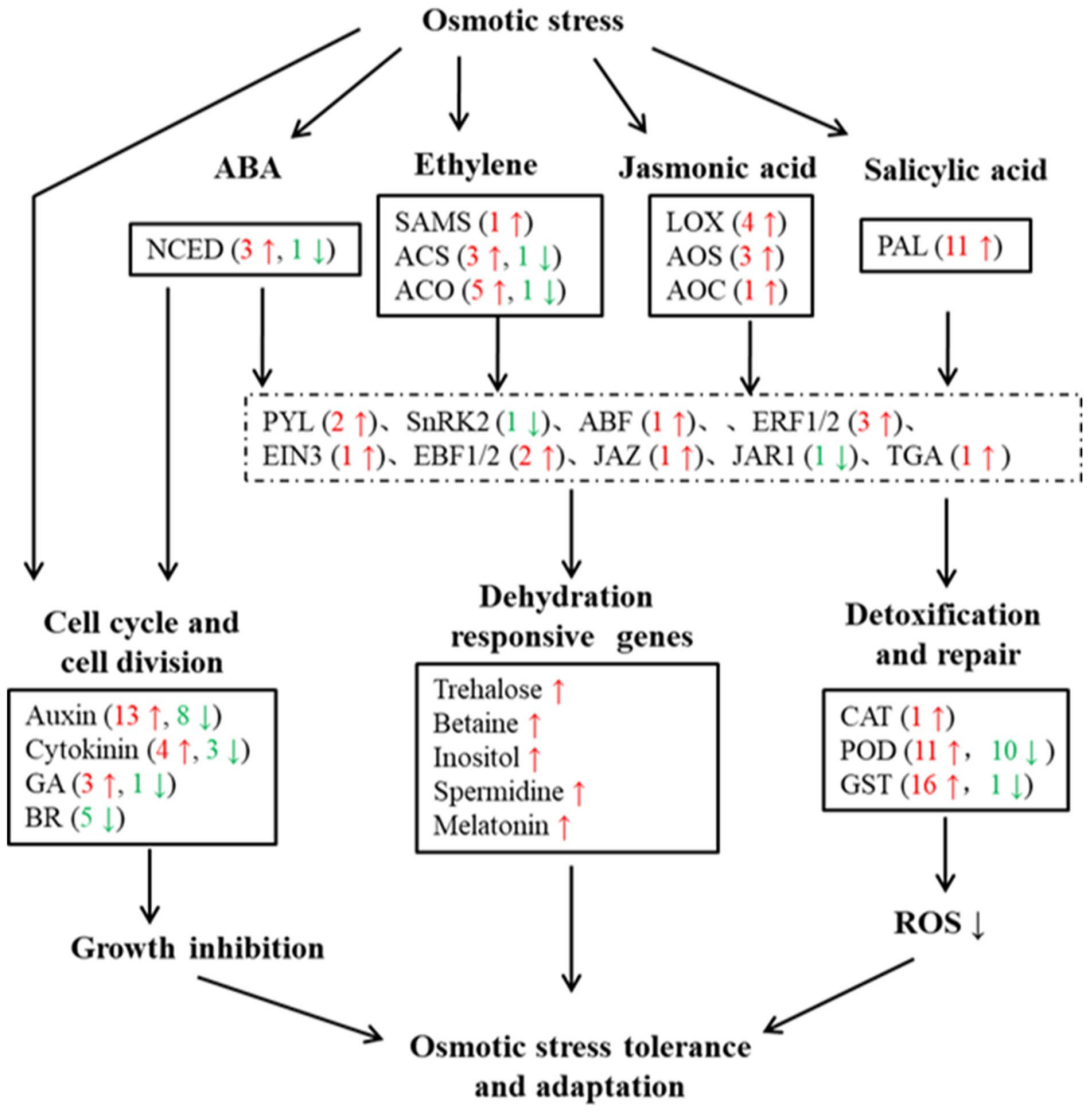
Overview of DEGs under osmotic stress in watermelon cells. Red arrows and numbers represent the number of up-regulated expressed genes and green arrows and numbers represent the number of down-regulated expressed genes.

To investigate the molecular mechanism of watermelon cells in response to osmotic stress, the expression pattern of DEGs was analyzed. The results showed that the expression levels of most genes related to cell growth changed dynamically, with 13 genes related to Auxin, 4 genes related to Cytokinin and 3 genes related to GA undergoing significant up-regulation of expression (Figure 2), suggesting that cell proliferation may be accelerating in response to adversity in the early stages. Meanwhile, the expression of genes related to plant hormone synthesis, signal transduction, osmoregulatory substance synthesis, and antioxidant system was up-regulated (Figure 2 and Supplementary Figure S1–S3) to maintain cellular homeostasis and accelerate the production of plant hormones such as ABA, JA, ETH and SA (Supplementary Figure S1F), which can act as signals to mediate downstream genes in response to osmotic stress and accelerate osmoregulatory substances to maintain osmotic pressure. This shows that the resistance response of watermelon to osmotic stress involves the synergistic changes of many biological processes directly and indirectly related to the resistance response.

### Methylation Landscapes in Watermelon

The bisulfite-transformed DNA of watermelon cells was treated with osmotic stress and control, sequenced with Illumina Hi-Seq in the coverage area, providing information on single cytosine methylation status and has a high degree of confidence. The results of WGBS are listed in Table 2. We obtained 60,344,781 and 63, 675, 83 reads after osmotic stress treatment and control. In addition, the mapping rate was 69.73 and 69.84%, respectively, showing that the method and results have high reliability and accuracy.

**Table 2:** Data summary of whole genome bisulfite sequencing.

Samples	Total reads	Mapped reads	Mapping rate (%)	Mean C (%)	Mean CG (%)	Mean CHG (%)	Mean CHH (%)
CK	63,767,583	44,513,056	69.84	25.47	60.51	39.62	19.32
OS	60,344,781	42,099,857	69.73	24.50	59.27	38.65	18.39

*Total reads are the clean sequencing reads after the data filtration. Mapping rate are the reads with unique positions on reference genome after alignments. Mean C, Mean CG, Mean CHG, and Mean CHH percentages (%) are the average methylation levels in the context of all C sites, CG, CHG, and CHH in the genome*.

**Figure 3:**
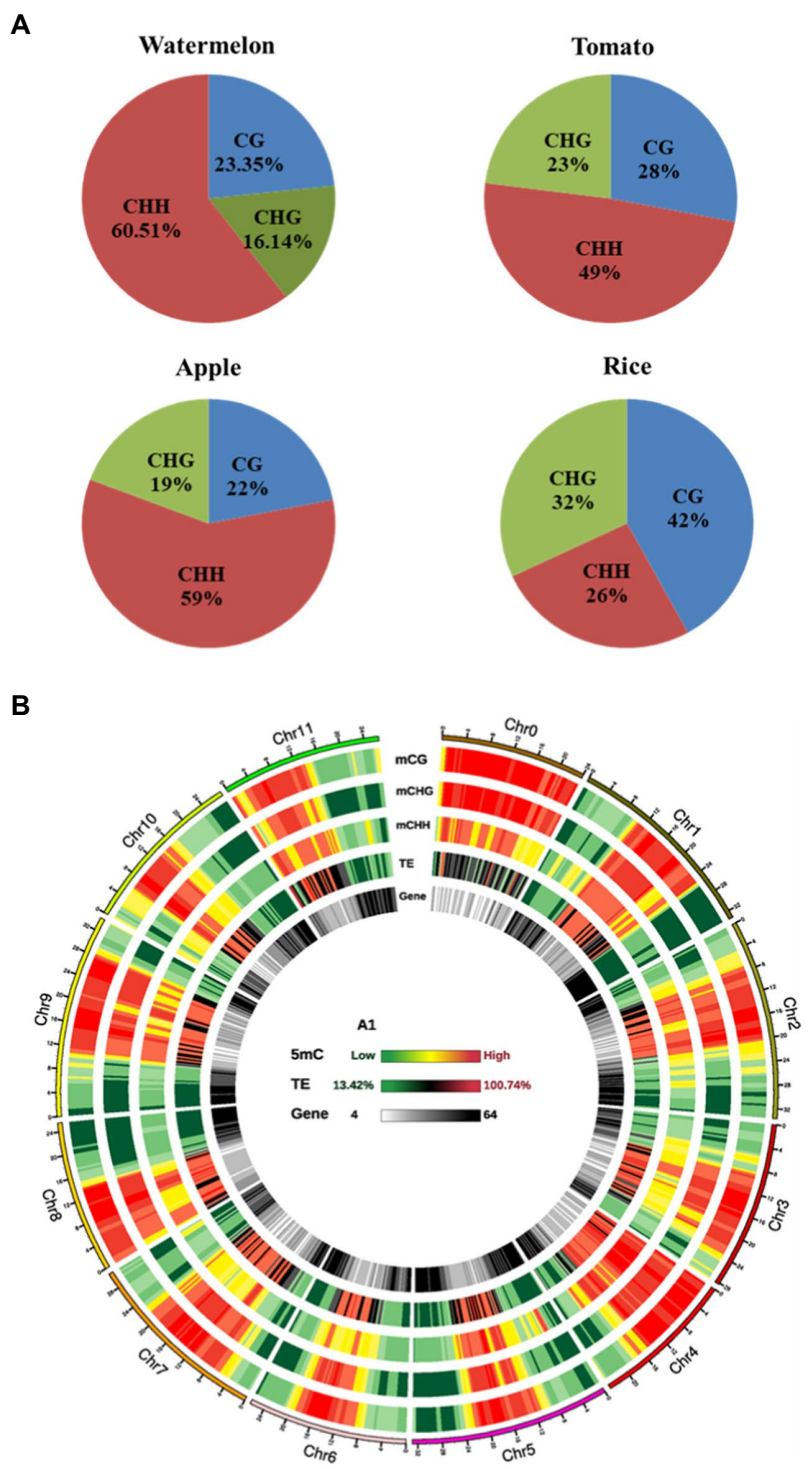
The watermelon epigenome. **(A)** Relative proportions of mC in three sequence contexts of different species. **(B)** Circos plot of watermelon chromosomes. Track order: density plot of mC in CG, CHG and CHH contexts; density of TEs; gene density of each chromosome. Red represents hypermethylation while green represents hypomethylation.

To analyze the changes of genome-wide methylation levels in control and osmotic stress treatments, we calculated the methylation levels of all C sites, CG, CHG, and CHH contexts in the genome (Table 2). The highest level of methylation occurred at the CG contexts, indicating that the CG contexts is the main methylation contexts in watermelon, followed by the CHG and the CHH contexts, which may be due to the CG contexts methylation is the most abundant type of DNA methylation (Stroud et al., 2014). Interestingly, it could be found that CG, CHG, and CHH contexts all exhibited hypomethylation patterns under osmotic stress. Compared with the control, the overall methylation level decreased by 0.96% after osmotic stress. And the methylation level in the CG, CHG, and CHH contexts decreased by 1.23, 0.96, and 0.93%, respectively. In addition, the proportion of mCG, mCHG, and mCHH on the total mC sites was calculated, which reflects the distribution of mCs in the three sequence contexts. As shown in Figure 3A, mC was most frequently found at the CHH contexts (60.51%), while it was less frequent in CG and CHG contexts (23.35 and 16.14%, respectively). Interestingly, there were differences in methylation level and methylation sites among different plant varieties (Figure 3A), speculating that methylation levels may be related to plant resistance and that watermelon may respond to osmotic stress by altering methylation levels and status.

On an overall scale, the distribution of DNA methylation on the whole chromosome was similar in the control and osmotic stress treatment, as represented by the Circos plot of the chromosomes of watermelon cells treated with osmotic stress for 4h (Figure 3B). DNA methylation was observed mostly on chromosome 0, and the high methylation levels were mainly concentrated in the middle of the chromosome and the low methylation levels were mainly concentrated at the ends of the chromosome. Furthermore, high methylation was found in TE-rich regions of watermelon genome, which was accompanied by low gene density. In contrast, TE-poor regions with low TE density were characterized by reducing methylation levels accompanied by higher gene density, we hypothesize that the main function of DNA methylation is to maintain genome stability.

### Association of DNA Methylation With Gene Expression

**Figure 4:**
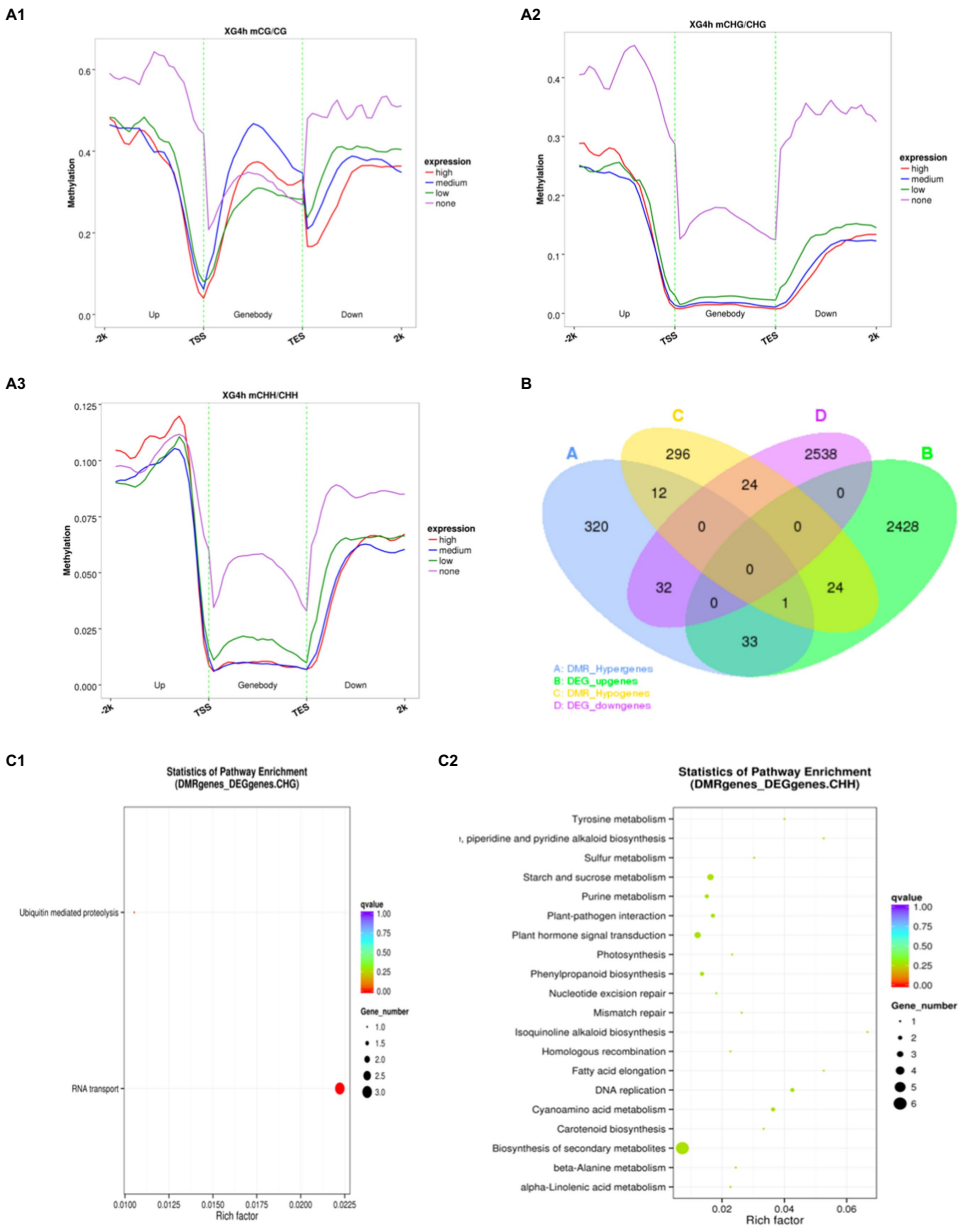
Methylation and gene expression association analysis. **(A)** The relationship between methylation level and gene expression. **(B)** Venn diagram of DEGs and DMGs. **(C)** KEGG pathway enrichment of DEMGs.

Dynamic changes in DNA methylation play an important role in the regulation of gene expression in plants in response to adversity (Sahu et al., 2013; Kim et al., 2015). To further investigate whether the changes in methylation levels triggered by osmotic stress are correlated with gene expression, the association analysis was carried out with transcriptome sequencing. These genes were classified into four categories based on expression levels: none expression levels, low expression levels, medium expression levels and high expression levels. Since the 2-h and 4-h samples showed a similar relationship between methylation and expression levels, the 4h_1 sample was used as an example to illustrate this relationship during osmotic stress response in watermelon cells (Figure 4A). It was found that the methylation level of unexpressed genes was significantly higher than other expressed genes, suggesting that gene silencing may be caused by high methylation (Figure 4A). The unexpressed genes showed high methylation levels in the upstream 2kb and downstream 2kb regions under all three contexts, and interestingly, lower methylation levels were observed near both TSS (transcription start site) and TES (transcription end site). For expressed genes, showed high CG methylation levels throughout most of the genebody, but relatively low methylation near the TSS and TES (Figure 4A1). In contrast, high expression corresponded to low CHG and CHH methylation levels, indicating CHG and CHH methylation levels were negatively correlated with expression in most regions of the genebody (Figure 4A2 and 4A3). Specifically, 114 genes were identified that showed both differential expression and differential methylation (Supplementary Table S2), which were defined as DMEGs (differentially methylated expression genes). 57 of these genes had transcriptional expression levels opposite to the methylation level, and another 57 had the same change in transcriptional and methylation levels, suggesting a possible correlation between DNA methylation and gene expression (Figure 4B). In addition, to deeply analyze the mechanism of methylation-regulated gene expression in response to osmotic stress, KEGG enrichment analysis was performed on DMEGs (Figure 4C). The results showed that DMEGs mostly occurred in CHH and CHG contexts and were mainly involved in RNA transport, plant hormone signal transduction, Biosynthesis of secondary metabolites, and starch and sucrose metabolism, suggesting that altered DNA methylation in watermelon may regulate the expression of genes involved in several important pathways in response to osmotic stress.

### Widespread Dynamic DNA Methylation in Response to Osmotic Stress

**Figure 5:**
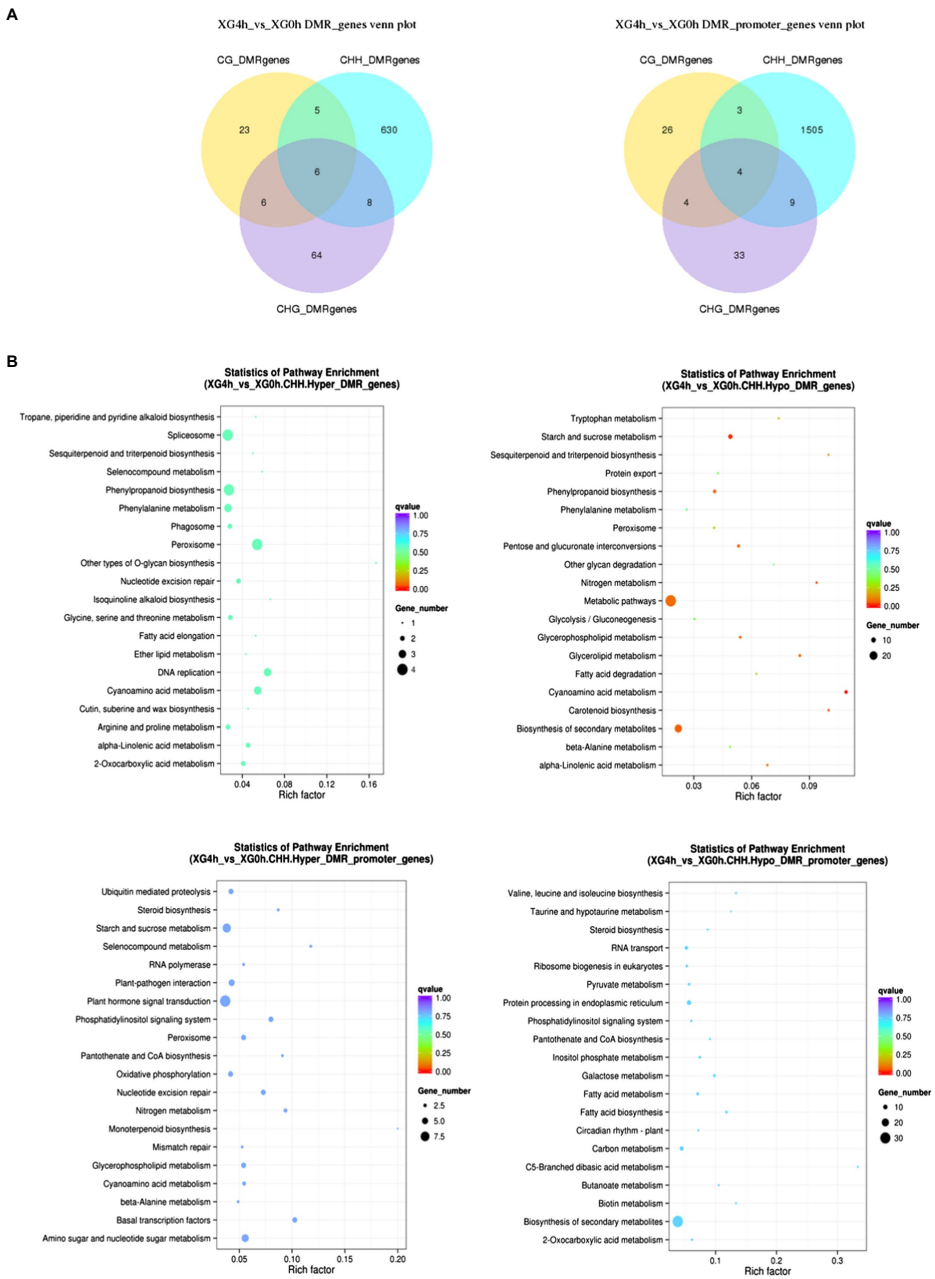
Analysis of differentially methylated genes under osmotic stress. **(A)** Venn diagram between DMGs in different contexts. **(B)** KEGG enrichment pathway of DMGs in CHH contexts.

To investigate the potential effect of osmotic stress on methylation, we analyzed DMGs (differentially methylated genes) between 4h of stress treatment and 0h of control. Figure 5A shows the identification of 742 and 1,584 DMGs in the genebody region and the promoter region, respectively, with the largest distribution in the CHH contexts with 649 in the genebody region and 1,521 in the promoter region, and fewer DMGs in the CG and CHG contexts with 40 and 84 in the genebody region and 37 and 50 in the promoter region, respectively. It indicates that the dynamic changes in methylation level of watermelon genome caused by osmotic stress mainly occurred at CHH contexts in the promoter and genebody regions. To investigate the biological functions of DMGs, we performed KEGG (Kyoto Encyclopedia of Genes and Genomes) pathway enrichment analysis. As shown in Figure 5B, methylation upregulated genes were mainly enriched in plant hormone signal transduction (13), starch and sucrose metabolism (7) and amino sugar and nucleotide sugar metabolism (6), and hypomethylation genes showed enrichment in the biosynthesis of secondary metabolites (49) and metabolic pathways (28) pathways. These suggested that osmotic stress changes the methylation status of genes involved in the synthesis of secondary metabolites (Supplementary Figures S4) and may lead to the synthesis of specific secondary metabolites.

### DNA Methylation May Be Involved in the Synthesis of Inositol Response to Osmotic Stress

**Figure 6:**
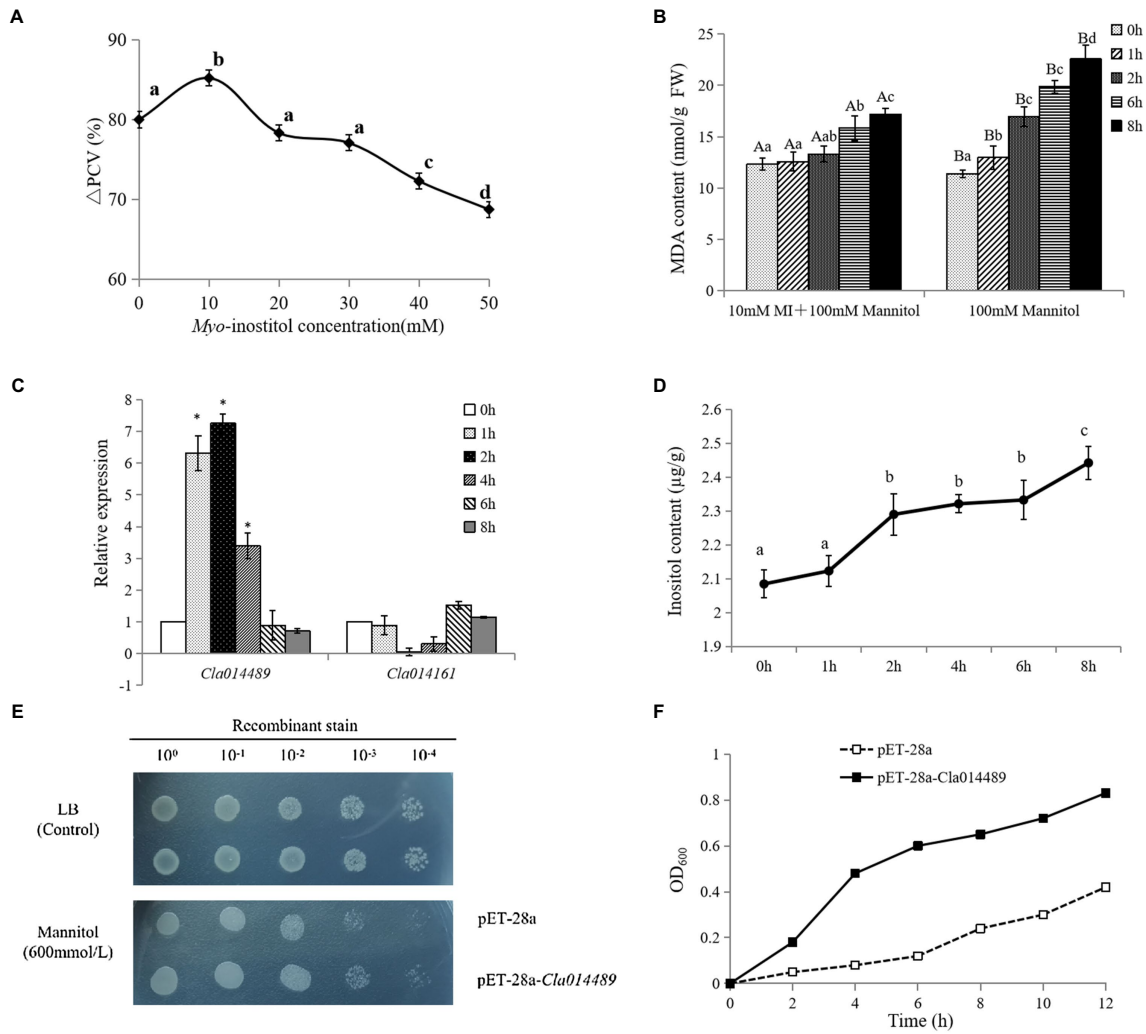
Regulation of osmotic stress by exogenous inositol in watermelon cells and analysis of *Cla014489* prokaryotic expression. **(A)** Effect of exogenous inositol on the growth of watermelon cells under osmotic stress. **(B)** Effect of exogenous inositol on the MDA content of watermelon cells under osmotic stress. **(C)** The expression of *Cl*MIPS in response to osmotic stress. **(D)** Inositol accumulation in watermelon cells under osmotic stress. **(E)** Droplet plate experiment of recombinant strain pET-28a-*Cla014489* and control strain pET-28a under osmotic stress. **(F)** Time course of growth of the recombinant strain pET-28a-*Cla014489* and the control strain (pET-28a) under 600mmol/l mannitol. Different uppercase and lowercase letters mean differences at *p*<0.05 and *p*<0.01 levels. Values are means ± SD of three biological replicates. * Indicates significantly difference (*p*<0.05) between the data point and 0 h data point.

Based on the analysis of changes in the content of osmoregulatory substances and expression of related genes (Supplementary Figures S3, S4), it was hypothesized that trehalose, betaine and inositol may play a key role in the response of watermelon cells to osmotic stress. Inositol is the one we want to focus on due to its less studied role in osmotic stress and the simplicity of the key enzyme gene family members. The alleviating effect of inositol on osmotic stress of watermelon cells was studied by measuring PCV and MDA content. The results showed that 10mM inositol alleviated the inhibition of cell growth by osmotic stress (Figure 6A) and protected the cell membrane structure possibly by reducing the accumulation of MDA (Figure 6B). In addition, it was found that osmotic stress induced up-regulated expression of *ClMIPS1* (*Cla014489*) during the first 4h of treatment (Figure 6C), and promoted the accumulation of inositol (Figure 6D). To verify the possible function of this gene, it was transferred into *E. coli* BL21 for prokaryotic expression and found that the expression of *Cl*MIPS protein improved the tolerance of *E. coli* against osmotic stress (Figures 6E and 6F), speculating that this gene may play an important role as a resistance gene in watermelon in response to osmotic stress.

**Figure 7:**
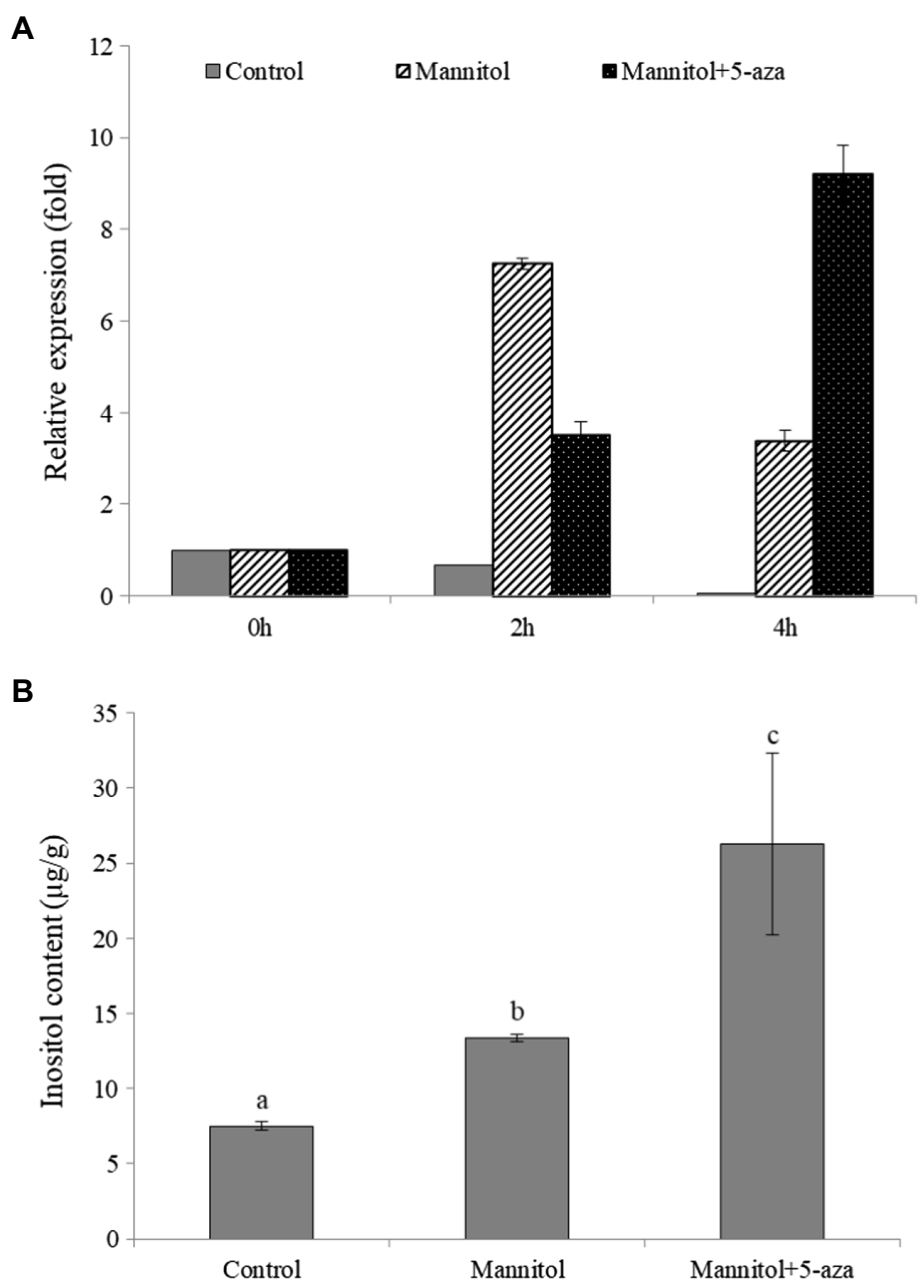
The osmotic regulation of inositol on watermelon cells may be associated with DNA methylation. **(A)** The effect of different treatments on the expression of *ClMIPS1*. **(B)** The effect of different treatments on the accumulation of inositol. Different lowercase letters mean significant differences (*p*<0.05). Values are means ± SD of three biological replicates.

To further investigate whether DNA methylation is involved in the regulation of inositol-related genes, 5-azacytidine (5-aza), a DNA methyltransferase inhibitor was added to, analyze its effect on inositol content as well as *ClMIPS1* gene expression. The results showed that *ClMIPS1* exhibited a higher expression at 4h of stress after pretreatment with the methylation inhibitor than at 2h of stress without the inhibitor treatment (Figure 7A), and the inositol content was significantly higher after mannitol and the addition of 5-aza treatment than the control (Figure 7B). It indicated that inhibition of DNA methyltransferase increased the expression of *ClMIPS1* and the accumulation of inositol in watermelon cells in response to osmotic stress.

## Discussion

Although stress expression patterns in watermelon in response to water stress have been explored in roots and leaves (Wang et al., 2014; Yang et al., 2016), the resolution of watermelon adversity regulation and stress response mechanisms requires more refined and comprehensive studies to characterize the molecular mechanisms of watermelon response to osmotic stress. Single-cell transcriptome sequencing technology can exclude expression heterogeneity among different cells and obtain a global map of gene expression within a single cell, thus helping to resolve the resistance mechanisms of plant interactions with abiotic stresses, and is a powerful tool currently used to study the mechanisms of plant stress resistance (Anderson and Mitchell-Olds, 2011; Li et al., 2013). Suspension culture cell is an important model system for studying *in vitro* cellular level responses to biotic and abiotic stresses in recent years; moreover, they have been utilized to obtain early response signals to stress and are now widely used in biochemistry, cytology, physiology, and molecular biology research fields (Pech-Kú et al., 2017; Li et al., 2018). Based on the previous analysis on the expression characteristics of key synthase genes of osmoregulatory substances such as trehalose, betaine and spermine, a stable watermelon suspension cell culture system was established (Xu et al., 2018b), most of them showed significant changes in expression at first few hours. Here, by transcriptome and whole-genome sequencing of established watermelon suspension cell line, we found that the methylation dynamics of the genome was closely related to the response of watermelon to osmotic stress and that the osmotic stress-induced decreased in the methylation level of the inositol synthase gene *Cl*MIPS is one of the mechanisms by which watermelon cells respond and cope with osmotic stress.

### Complex Regulatory Mechanisms of Watermelon Cells in Response to Osmotic Stress

The response of plants to adversity stress involves multiple processes such as biosynthesis, translocation, ion permeation, substance metabolism and signal transduction, which require the involvement of multiple genes and transcription factors (Wicker et al., 2006). GO functional annotation revealed that watermelon cells DEGs were most enriched in molecular functions (MF), with highly significant up-regulated genes in transcription factor activity, DNA binding and transmembrane transporter protein activity (Figure 1C), including salicylic acid responsive transcription factor (*TGA*), ethylene responsive transcription factor (*ERF*) and abscisic acid responsive transcription factor (*ABF*), which are related to plant adversity. These transcription factors can initiate the expression of downstream genes, synthesize plant hormones, osmoregulatory substances and related protective proteins, and mitigate the damage of cells by osmotic stress. In biological processes, genes that maintain intra- and extracellular homeostasis, such as ion transport, transmembrane transport and cation transport, were up-regulated in expression. Meanwhile, the up-regulated expression of genes related to cell growth such as Auxin, Cytokinin, and GB was inconsistent with the response of watermelon roots to drought stress (Wang et al., 2014). We hypothesized that cells accelerated their proliferation rate during the pre-response to osmotic stress. However, with the increase of stress time, the cell accumulation decreased, growth was inhibited, and growth rate slowed down, which is consistent with the conclusion reached by our previous authors (Xu et al., 2018a). It is hypothesized that watermelon responds to adversity stress by slowing growth rate and reducing energy expenditure during long-term environmental stress. The KEGG enrichment analysis revealed that the DEGs of watermelon cells in response to osmotic stress were mainly involved in antioxidant systems including glutathione metabolism, ascorbic acid metabolism and synthesis; amino acid biosynthesis, metabolism, and glycolysis related to osmoregulatory substances; and calcium channels and plant hormone signals transduction pathways (Figures 1B,C). Complex regulatory mechanisms of watermelon cells in response to osmotic stress were hypothesized based on DEGs enriched in these metabolic pathways: cell growth-related genes in cells were down-regulated and cell growth rate was inhibited. At the same time, the expression of genes related to plant hormones synthesis, signal transduction, osmolyte synthesis, antioxidant system and ion transport was up-regulated to maintain cellular homeostasis and accelerate the production of plant hormones such as ABA, JA, and SA, which can act as signals to mediate downstream genes in response to osmotic stress. Thus, it can be seen that the resistance response of watermelon to osmotic stress involves the synergistic changes of many biological processes directly and indirectly related to the resistance response (Kazuko and Kazuo, 2006). There is growing evidence implicating epigenetic mechanisms such as DNA methylation in regulating gene expression to modulate plant responses to environmental stress (Boyko and Kovalchuk, 2011). Liang et al. (2014) found that transcription factors undergo demethylation in response to drought stress in *populus trichocarpa*. Xu et al. (2015) revealed that the salt-induced transcription factor *MYB74* is regulated by the RdDM pathway in *Arabidopsis*. Similarly, Lu et al. (2017) revealed that upland cotton undergoes demethylation of phytohormone-related genes in response to drought stress. These findings suggested the existence of dynamic regulation of DNA methylation in plant abiotic stress response, but the dynamic changes in DNA methylation characterized in watermelon have not been reported.

### Dynamic DNA Methylation Under Osmotic Stress

An increasing number of studies have shown that epigenetic mechanisms play an important role in plant responses to abiotic stress, which makes comparative epigenomics becoming a new area of research (Wang et al., 2016). In this study, we mapped the single-base methylomes of watermelon by WGBS with methylation levels of 60.51, 39.62, and 19.32% at CG, CHG, and CHH contexts, respectively (Table 2). Based on previous methylation studies, CG sequence environmental methylation showed the highest levels in various species ranging from 30.5% in *Arabidopsis* to 92.5% in *Beta vulgaris*; CHG methylation varied from 9.3% in *Eutrema salsugineum* to 81.2% in *Beta vulgaris*, and CHH methylation varied from 1.1% in *Vitis vinifera* to 28.5% in *Raphanus sativus* (Niederhuth et al., 2016), speculating that DNA methylation levels in plants of different species may be related to genome size, stress resistance, and environmental factors (Ausin et al., 2016; Li et al., 2020). Interestingly, the overall methylation level of the watermelon genome was down-regulated after osmotic stress, and all three contexts, CG, CHG, and CHH, showed a demethylation pattern after stress treatment, which is consistent with previous studies (Liang et al., 2014; Lu et al., 2017; Xu et al., 2018a). There are growing evidences that methylation and demethylation processes are always associated with alterations in gene expression. Therefore, understanding the dynamic changes of epigenetic DNA methylation modifications in plants under environmental stress will help to study functional gene expression and further elucidate the molecular mechanisms of plant response to adversity. Lu et al. (2017) found that altered methylation status changed the transcription and expression of hormone-related genes, and in particular, inhibition of methyltransferases enhanced drought resistance in upland cotton. Xu et al. (2018a), a study in apple against water stress, found that genes with unmethylated promoters exhibited higher expression levels than genes with methylated promoters, and genebody methylation appeared to be positively correlated with gene expression. Wang et al. (2016) observed fewer DMR in drought-tolerant rice than in sensitive rice, implying that DNA methylation is more stable in drought-tolerant genotypes. Interestingly, we found that the overall methylation level of the watermelon genome was down-regulated after osmotic stress, with three contexts, CG, CHG, and CHH, showing a demethylation pattern after stress treatment. Although the decrease was less pronounced, it may be due to the more stable genome of watermelon as a species originating from arid regions.

The regulatory role of DNA methylation on plant gene expression has been extensively studied, and there is growing evidence that methylation levels are consistently associated with altered gene expression. It is generally accepted that hypermethylation prevents the binding of transcription factor complexes to DNA to repress gene expression, whereas hypomethylation facilitates gene expression (Chan et al., 2005; Zilberman et al., 2007; Li et al., 2012). In this study, comparison of methylome and transcriptome in watermelon cells before and after osmotic stress treatment showed that the relationship between DNA methylation levels and gene expression levels was more subtle than initially realized and that there was no direct linear relationship. These genome-scale methylation analyses provide new insights into the regulation of DNA methylation on phenotype. In addition, the methylation changes in the CHH contexts were more significant than those in the CG and CHG contexts, and the promoter region was more abundant in genes with altered methylation levels (Figure 5A). Zhong et al. (2013) demonstrated that DMRs induced by environmental stress were associated with promoter regulatory regions and inferred that the promoter region of CHH contexts hypomethylation may be a key regulatory mechanism in watermelon cells in response to osmotic stress. Correlation analysis of DEGs together with DMGs in response to osmotic stress indicated that watermelon cells may respond to osmotic stress by altering RNA transport, plant hormone signals transduction, biosynthesis of secondary metabolites, and methylation levels of genes involved in starch and sucrose metabolic pathways to regulate gene expression of this process. Because the expression pathways affected by DNA methylation are complicated, the important regulatory mechanisms involved in epigenetic modification of gene expression networks in the perception and response to adversity stress still need further elucidation.

### *ClMIPS1* Hypomethylation May Be a Key Regulatory Factor in Watermelon in Response to Osmotic Stress

Abiotic stress leads to an increase in oxygen radicals, and one of the toxic effects of excess free radicals is to cause membrane lipid peroxidation, which damages the membrane system and causes oxidative damage (Haupt-Herting and Fock, 2002). The results of previous experiments demonstrated that osmotic stress has a significant inhibitory effect on the growth of watermelon cells (Xu et al., 2018b). The results of this experiment revealed that exogenous addition of 10mM inositol treatment significantly alleviated the growth inhibition of watermelon suspension cells under osmotic stress (Figure 6A) and significantly reduced the MDA content in cells (Figure 6B), which may be related to the reactive oxygen species scavenging effect of inositol (Loewus and Murthy, 2000). It has been shown that inositol can act as an osmotic substance in osmoregulation, promoting water retention in the cytoplasm and allowing the entry of sodium ions into the vesicles or plasmodesmata (Knight and Knight, 2012). In addition, it can protect cellular structures from reactive oxygen species by controlling swelling pressure through interactions with membranes, protein complexes or enzymes (Bohnert et al., 1995). Essentially, inositol and its derivatives have complex mechanisms to regulate plant stress responses and are still the focus of active research (Valluru and Ende, 2011). Currently, there is no clear report on the mechanism of inositol as a small molecule osmoregulatory substance for the alleviation of osmotic stress. From this experiment, it can be tentatively inferred that exogenous inositol can protect cell membrane structure from reactive oxygen species by reducing or slowing down MDA production and maintaining or increasing antioxidant enzyme activity in plants.

MIPS is a key rate-limiting enzyme in the process of inositol synthesis, the only known catalase involved in the conversion between glucose-6-phosphate and inositol phosphate, and a prerequisite substance for many compounds involved in signal transduction, phosphorus storage, phytohormone homeostasis, stress response, and cell membrane and cell wall synthesis in plants (Loewus and Murthy, 2000). In contrast to animals, multiple *MIPS* genes are present in plants, with seven found in maize, three in *Arabidopsis*, and four in soybean, and multiple *MIPS* genes in plants can be applied to regulate differential expression into specific physiological functions. In this study, two MIPS-encoding genes were identified from watermelon cells and their expression characteristics were analyzed under osmotic stress, and it was found that osmotic stress only induced *Cla014489* expression, while *Cla014161* expression was repressed (Figure 6C). Analysis of their promoter region revealed *cis*-elements responded to hormones such as ETH, SA and MeJA (Supplementary Figure S5A), which were presumed to be inducible promoters. In this study, exogenous ABA, ETH, MeJA and SA were used to induce the expression of *Cl*MIPS genes and discovered that the expression of *Cla014489* increased in different degrees after the four plant hormones induction treatments, with the changes more obvious after MeJA and ETH treatments, it was speculated that JA and ETH might be the intermediate signals to induce this basic expression. In contrast, the induction of expression of *Cla014161* gene by ETH and MeJA was not obvious, and the expression level of this gene changed significantly after SA and ABA treatment, and it was inferred that SA and ABA may be the intermediate signals to induce the expression of this gene.

**Figure 8:**
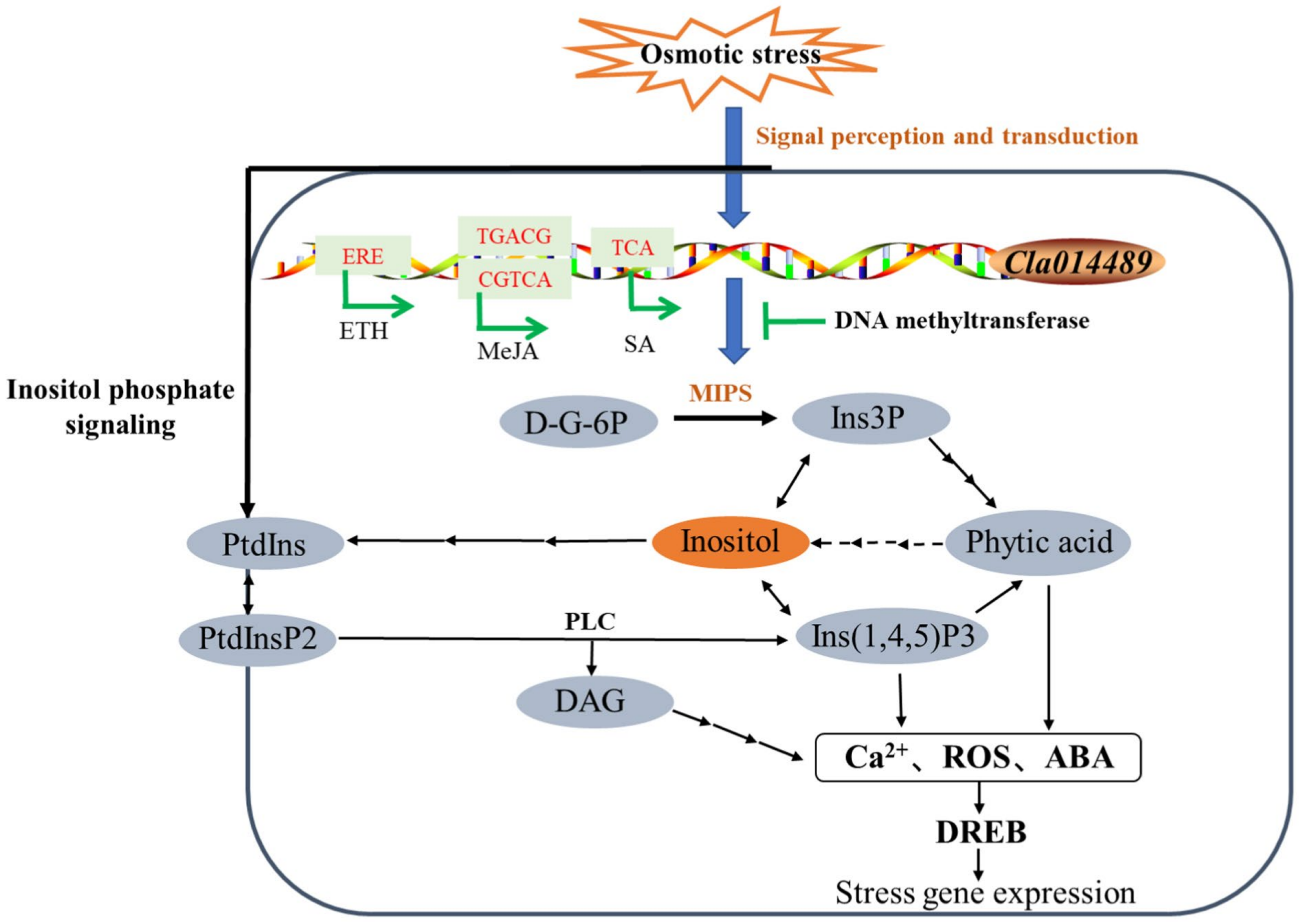
Model of regulation of *Cl*MIPS in response to osmotic stress. “→” represents induction of phytohormone, while “⊥” stands for suppression. Solid lines indicate identified pathways and dashed lines indicate speculative pathways.

Currently, the genes encoding MIPS have been isolated from a variety of plants, and Majee et al. (2004) produced transgenic lines with salt tolerance by overexpressing the MIPS-encoding gene *PINO1* in tobacco. In this experiment, only *Cla014489*, which has inositol-1-phosphate synthase activity and is involved in inositol biosynthesis, was induced to be expressed by osmotic stress. Thus, we investigated the function of this gene, and we successfully cloned *Cla014489* from watermelon cells, which was found to have an ORF of 1,533bp and encodes 510 amino acids by sequencing analysis. The recombinant plasmid pET-28a-*Cla014489* was constructed and transformed into *E. coli* BL21, and the results showed that BL21 bacteria expressing *Cl*MIPS protein grew better than the control under 600mM mannitol treatment (Figures 6E,F). It suggests that this gene is closely related to stress adaption. The methylation inhibitor 5-aza can reduce DNA methylation levels by inhibiting DNA transferase activity. 5-aza pretreatment caused high expression of the inositol synthesis key enzyme gene which could respond to osmotic stress and inositol accumulation in watermelon cells (Figures 7A,B), and it is speculated that the decrease in methylation status of *ClMIPS1* is one of the mechanisms in response to osmotic stress (Figure 8). It can promote the accumulation of inositol through dynamic changes in methylation levels, the induction of various plant hormones such as ETH, JA and SA regulation, and even generate inositol or its derivatives through phosphatidylinositol signaling on the cell membrane to participate in the plant environmental stress response process together. It has been shown that external abiotic stress signals can be sensed and received through receptors or membrane receptors on the cell membrane, and rely on plasma membrane Ca^2+^ channels to transmit signals (Knight and Knight, 2012). Meanwhile, inositol and its metabolites play a key role in membrane synthesis, Na^+^ sequestration, and maintenance of photosynthetic capacity (Nelson et al., 1998). It is speculated that *Cl*MIPS, a key rate-limiting enzyme gene is a precursor substance for inositol synthesis, may maintain cellular osmotic pressure and reduce abiotic stress damage to plants by affecting cell membrane stability or absorbing higher concentrations of Na^+^ from the environment. Another study found that exogenous addition of inositol increased plant stress resistance probably because of the elevated content of inositol derivatives—phosphatidylinositol or inositol galactosides, which in turn caused changes in downstream substances (Zhai et al., 2016).

Due to the multiple important roles of inositol in plants and the complex regulation of epigenetics, it is hypothesized that its role in adversity is also complex and intertwined, and from the present work it appears that inositol may not act primarily or only as an osmoprotectant. To investigate in depth, the role of MIPS and inositol metabolism in osmotic stress, a genetic transformation system of watermelon for *Cl*MIPS overexpression studies needs to be established subsequently. In addition, inositol trisphosphate (IP_3_) is one of the dual messengers of the intracellular signaling system, and whether inositol regulates plant cells to maintain homeostasis by affecting the signaling system remains to be further explored.

## Data Availability Statement

The datasets presented in this study can be found in online repositories. The names of the repository/repositories and accession numbers can be found at: NCBI Sequence Read Archive (SRA, https://www.ncbi.nlm.nih.gov/sra/) under accession number PRJNA770012 and PRJNA770013.

## Author Contributions

XJ and FQ designed the experiments. FZ, ML, and MY performed the experiments. FZ, ML, and MY analyzed the data. FZ, MY, and FQ wrote the paper. All authors read and approved the final manuscript.

## Funding

This work was financially supported by the National Natural Science Foundation of China (No. 31760595) and Hainan Provincial Natural Science Foundation of China (No. 321RC473).

## Conflict of Interest

The authors declare that the research was conducted in the absence of any commercial or financial relationships that could be construed as a potential conflict of interest.

## Publisher’s Note

All claims expressed in this article are solely those of the authors and do not necessarily represent those of their affiliated organizations, or those of the publisher, the editors and the reviewers. Any product that may be evaluated in this article, or claim that may be made by its manufacturer, is not guaranteed or endorsed by the publisher.

## Abbreviations

5-aza: 5-azacytidine
DEGs: Differentially expressed genes
DMEGS: Differentially methylated expressed genes
DMGs: Differentially methylated genes
DMRs: Differential methylation regions
ETH: Ethylene
GA: Gibberellin
GO: Gene ontology
JA: Jasmonic acid
KEGG: Kyoto encyclopedia of genes and genomes
mC: Methylcytosine
MDA: Malondialdehyde
MeJA: Methyl jasmonate
PCV: Packed cell volume
SA: Salicylic acid
TES: Transcription end site
TSS: Transcription start site
WGBS: Whole-genome bisulfite sequencing.
