# Supplementary material for: Integrated Transcriptome Analysis and Single-Base Resolution Methylomes of Watermelon (Citrullus lanatus) Reveal Epigenome Modifications in Response to Osmotic Stress
Source: Front Plant Sci. 2021 Nov 29;12:769712. doi: 10.3389/fpls.2021.769712 (PMC8667863; doi:10.3389/fpls.2021.769712)
Supplement: Supplementary file 1 [file Data_Sheet_1.docx]

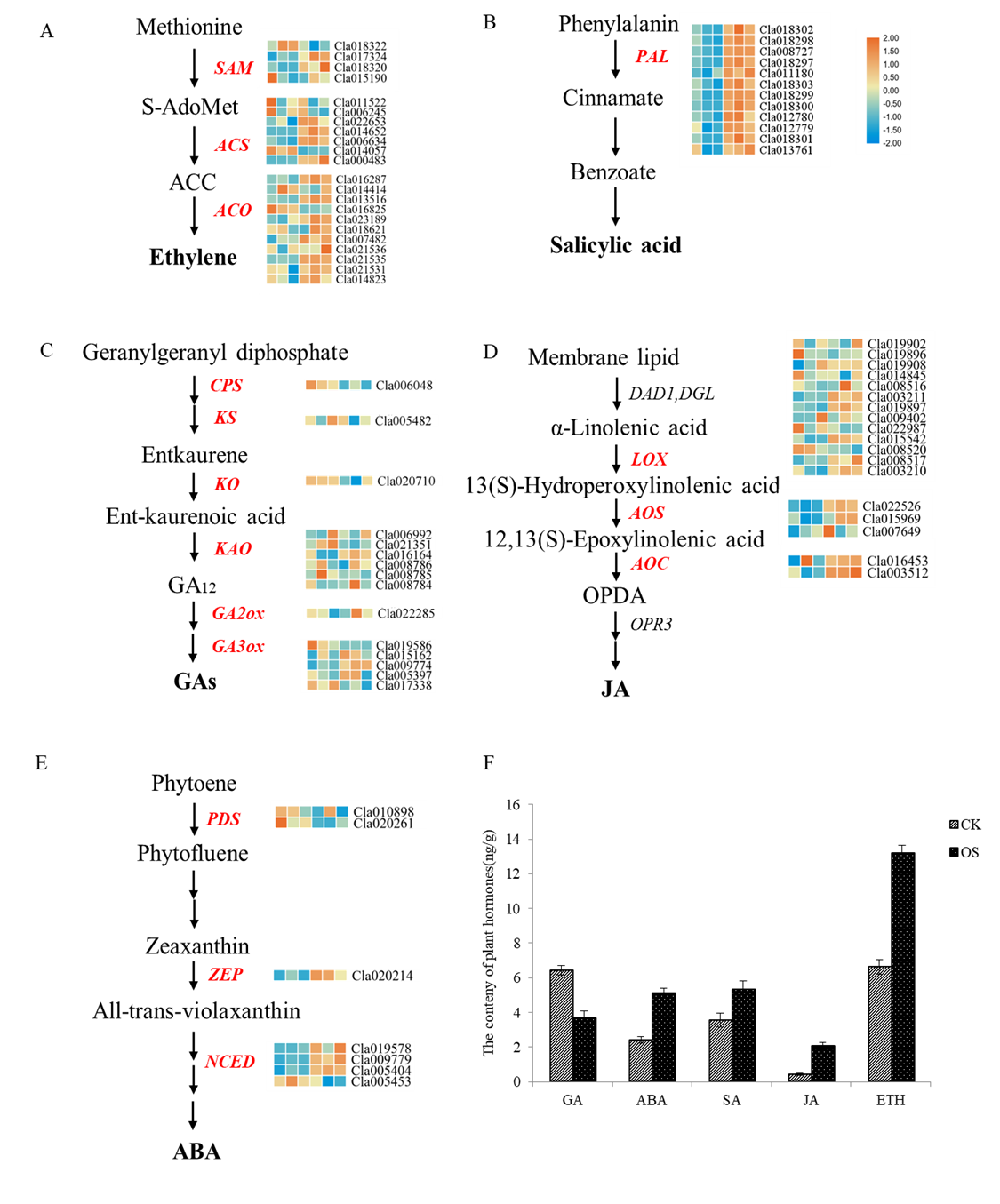


Figure S1. Changes in plant hormone content and synthase gene expression under osmotic stress in watermelon cells. (A) Ethylene synthesis-related genes; (B) Salicylic acid synthesis-related genes; (C) Gibberellin synthesis-related genes; (D) Jasmonic acid synthesis-related genes; (E) Abscisic acid synthesis-related genes; (F) Changes in phytohormone content under osmotic stress. The heat map from blue to red indicates the expression level of genes from low to high. The same below.


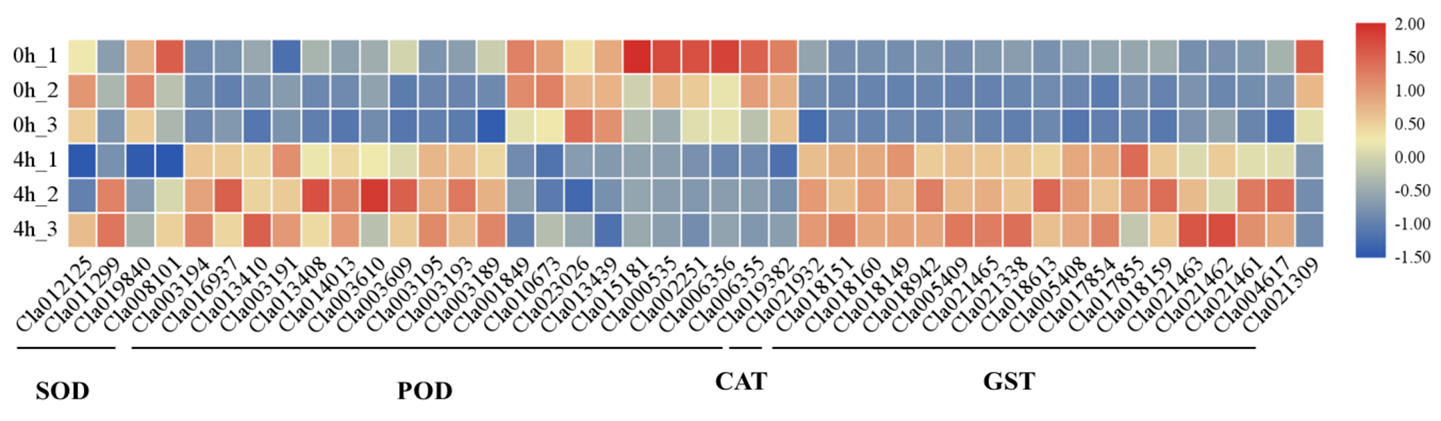


Figure S2. Expression levels of DEGs associated with reactive oxygen scavenging.


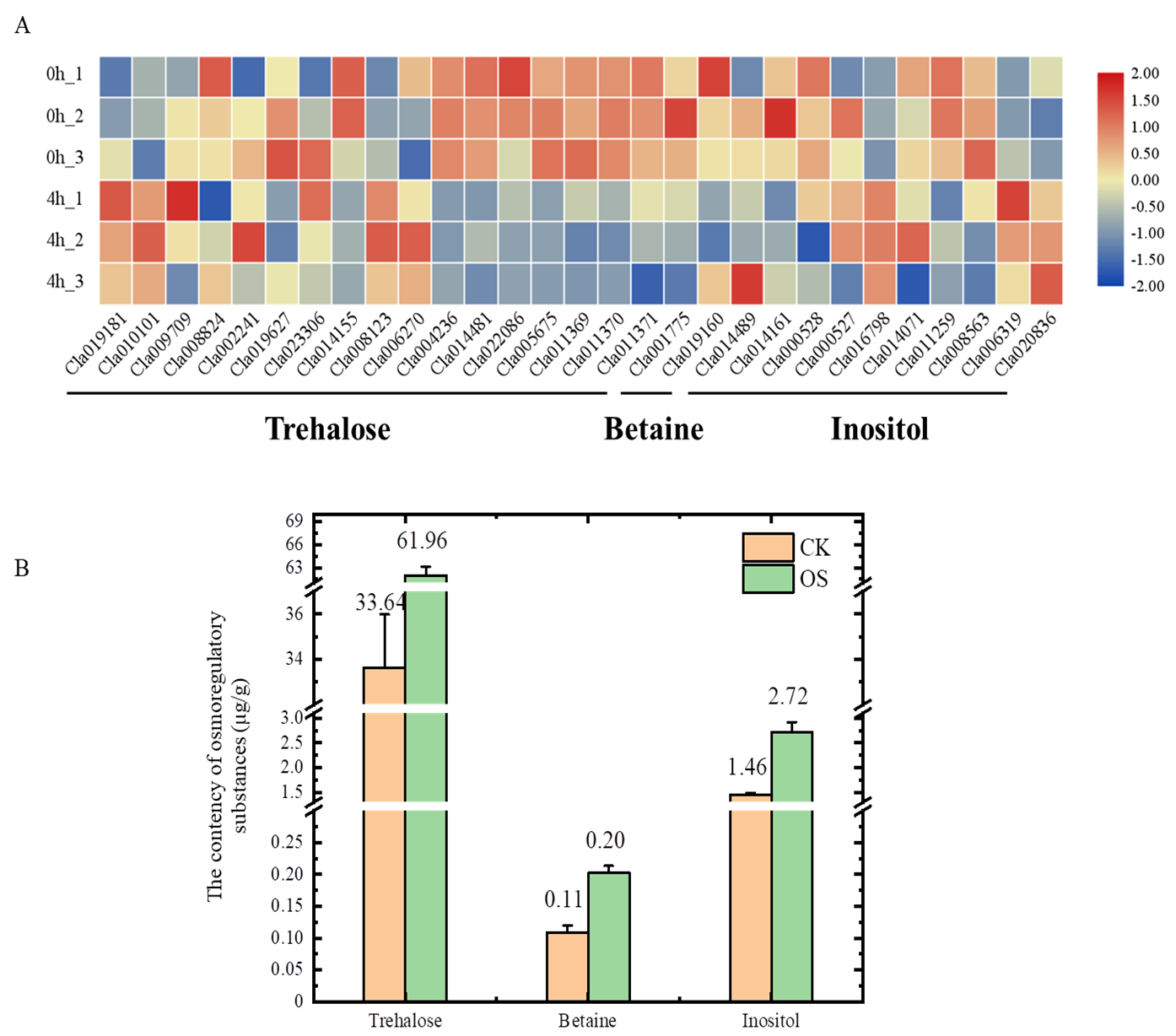


Figure S3. Changes in the content of osmoregulatory substances and expression of anabolic enzyme genes in watermelon cells under osmotic stress. (A) Heat map of expression of genes related to osmoregulatory substances; (B) Changes in osmoregulatory substances content under osmotic stress.


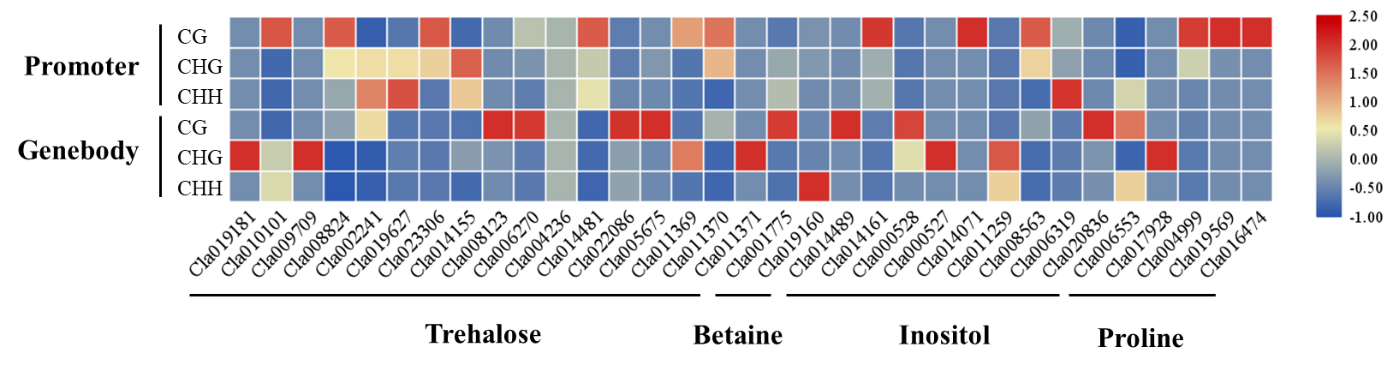


Figure S4. Changes in methylation of secondary metabolite anabolic genes associated with osmoregulation. Red indicate hyper methylation, blue indicate hypo methylation.


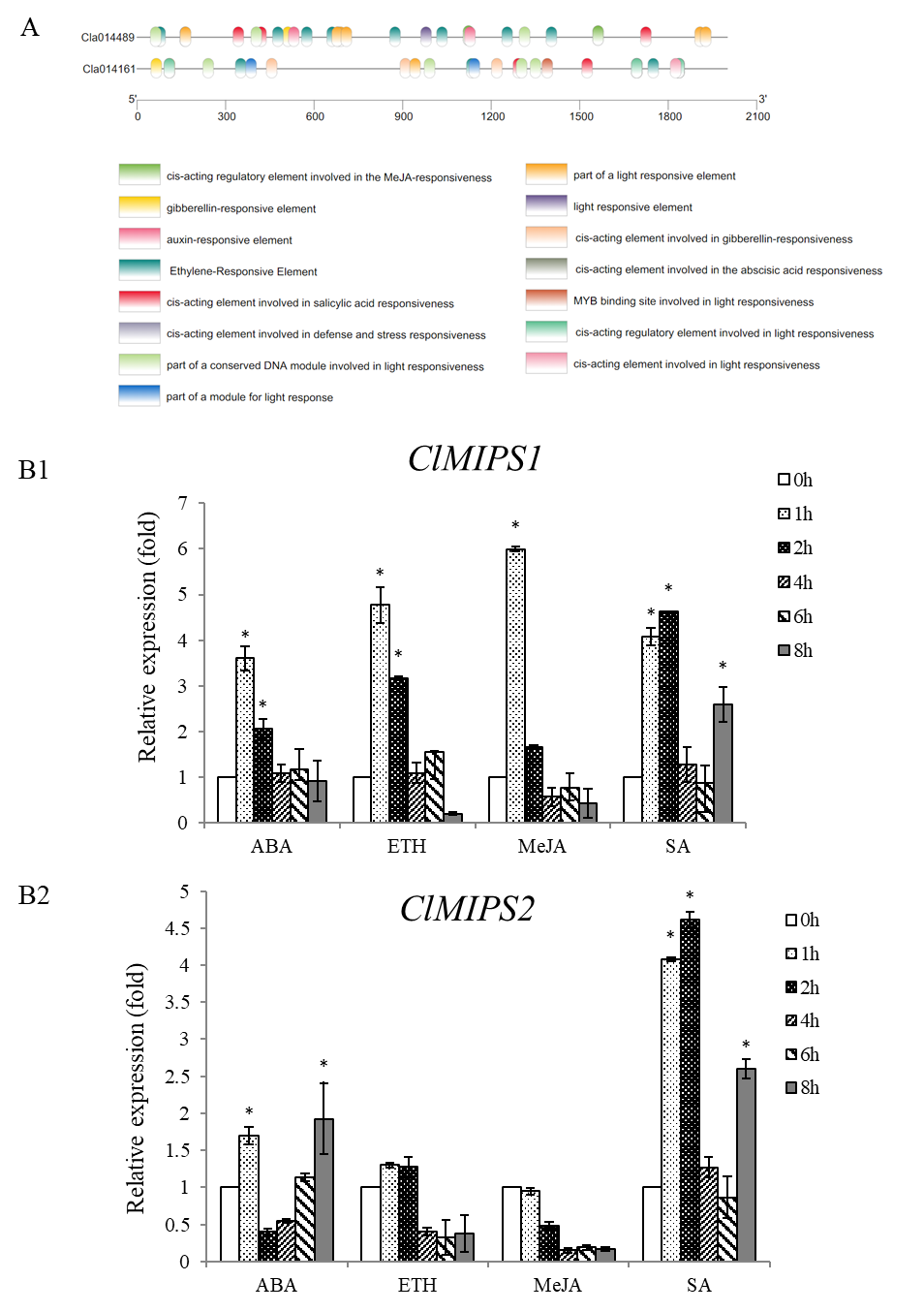


Figure S5 Expression characteristics of hormone induced *ClMIPS*. (A) Putative *cis*-regulatory elements identified in the promoter regions of *ClMIPS.* (B) The relative expression of *ClMIPS* induced by different hormones. * indicate statistical significant difference at confidence levels of *p* < 0.05.

Table S1. Primers used in this study.

| Primer name | Forward primer | Reverse primer |
| --- | --- | --- |
| qRT-PCR-*18s** | CATCCAAGGAAGGCAGCAGG | CAGACTCGAAGAGCCCGGTA |
| pET-28a-*Cla014489* | GTGCCGCGCGGCAGCCATATGTTCATCGACAGCTTTAAGGTCG | TTGTCGACGGAGCTCGAATTCTCACTTGTATTCCAAAATCATGTTGT |
| qRT-PCR-*Cla014489*  qRT-PCR-*Cla014161* | TCTCACTGGAGGTATCATAG  GGGAGCATCCAGACCATGTTGTAG | GATCTCCTCACCATCAATAG  GGGAGTCCTCGCATGTGTTGTG |

Note：The restriction site of endonuclease are shown in the box.
